# Root transcriptome profiling of contrasting wheat genotypes provides an insight to their adaptive strategies to water deficit

**DOI:** 10.1038/s41598-020-61680-1

**Published:** 2020-03-17

**Authors:** Md. Sultan Mia, Hui Liu, Xingyi Wang, Chi Zhang, Guijun Yan

**Affiliations:** 1 UWA School of Agriculture and Environment, Faculty of Science, The University of Western Australia, Perth, WA Australia; 2 The UWA Institute of Agriculture, The University of Western Australia, Perth, WA Australia; 3 Department of Plant Breeding, Bangladesh Agricultural Research Institute, Gazipur, Bangladesh; 4 Beijing Genomics Institute-Shenzhen, Shenzhen, 518083 China

**Keywords:** Gene expression, Drought

## Abstract

Water deficit limits plant growth and productivity in wheat. The effect of water deficit varies considerably in the contrasting genotypes. This study attempted comparative transcriptome profiling of the tolerant (Abura) and susceptible (AUS12671) genotypes under PEG-simulated water stress via genome-wide RNA-seq technology to understand the dynamics of tolerance mechanism. Morphological and physiological analyses indicated that the tolerant genotype Abura had a higher root growth and net photosynthesis, which accounted for its higher root biomass than AUS12671 under stress. Transcriptomic analysis revealed a total of 924 differentially expressed genes (DEGs) that were unique in the contrasting genotypes under stress across time points. The susceptible genotype AUS12671 had slightly more abundant DEGs (505) than the tolerant genotype Abura (419). Gene ontology enrichment and pathway analyses of these DEGs suggested that the two genotypes differed significantly in terms of adaptive mechanism. Predominant upregulation of genes involved in various metabolic pathways was the key adaptive feature of the susceptive genotype AUS12671 indicating its energy-consuming approach in adaptation to water deficit. In contrast, downregulation the expression of genes of key pathways, such as global and overview maps, carbohydrate metabolism, and genetic information processing was the main strategy for the tolerant genotype Abura. Besides, significantly higher number of genes encoding transcription factors (TF) families like MYB and NAC, which were reported to be associated with stress defense, were differentially expressed in the tolerant genotype Abura. Gene encoding transcription factors TIFY were only differentially expressed between stressed and non-stressed conditions in the sensitive genotype. The identified DEGs and the suggested differential adaptive strategies of the contrasting genotypes provided an insight for improving water deficit tolerance in wheat.

## Introduction

Wheat is one of the most important cereal crops in terms of area of cultivation and production throughout the world^1^. In Mediterranean environment, such as in Australia, a common feature for wheat production is that wheat is primarily grown under rain-fed condition^2^. Limited and erratic pattern of rainfall poses a high risk for successful crop production throughout their life cycle^3^. Available soil moisture plays a crucial role during early seedling growth for successful crop establishment in those areas. Water deficit during this stage limits crop growth and development. Several previous studies have demonstrated that plants when subjected to water deficit during the early stage, their root growth is restricted severely^4–8^.

Roots are the primary organs that perceive the signals of water deficit and produce responses at cellular and molecular such as genomic, transcriptomic and metabolic levels. Maintaining root growth under low water potential is considered as an effective adaptive response. Tolerant genotypes can maintain root growth at low water potential that enables them to maintain an adequate water supply. Therefore, root growth under water deficit is an effective indicator of plant adaptation and this has been exploited in trait-based drought-tolerance breeding programs for different crops^9–11^.

In general, plants when subjected to water deficit exhibit adaptive mechanism involving specific morphological, physiological and molecular processes^12–14^. It has been reported that these processes involve enhanced or reduced expression of related genes to compensate for the stress damage^15^. Some of those genes, such as dehydration-responsive-element-binding (DREB) genes, encode “effector protein” rendering direct defense whereas others act passively by encoding regulatory proteins in the form protein kinase and transcription factors that regulate the expression of stress-related genes^16,17^. With the advancement of next-generation sequencing technology and a dramatic reduction in associated cost, transcriptome profiling is a highly effective way to investigate the expression of stress-related genes and their related pathways. Comparative transcriptome profiling between contrasting genotypes has been studied to elucidate different molecular mechanisms of stress tolerance in various crops^18–22^. However, in wheat, most of those studies focused mainly on the water deficit tolerance at the later stage of the life cycle and many of them were accomplished using aboveground tissues^23–25^. In this study, next-generation transcriptome sequencing was applied to elucidate how water deficit causes significant changes in gene expression in roots of the two contrasting genotypes and to reveal the underlying mechanisms that play a crucial role at early growth stage water deficit tolerance. The two genotypes of bread wheat, Abura and AUS 12671, hereafter termed as ABU and AUS, were selected based on their contrasting performances (% root length reduction) under early-stage PEG-simulated water deficit from a previous screening experiment comprising of fifty genotypes of diverse origins^26^.

## Results

### Abura (ABU) and AUS12671 (AUS) differ in morphological and physiological traits under water deficit

**Figure 1:**
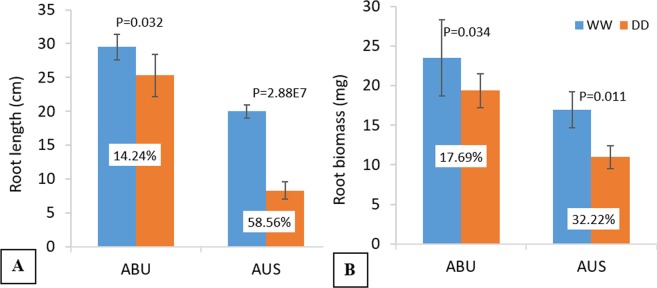
Effect of water stress on root morphology. (**A**) Root length of the contrasting tolerant genotype, Abura (ABU) and susceptible genotype, AUS12671 (AUS) under control (WW) and stressed (DD) conditions; (**B**) Root biomass of the contrasting genotypes under control (WW) and stressed conditions (DD). Values in white boxes show % reduction due to stress.

**Figure 2:**
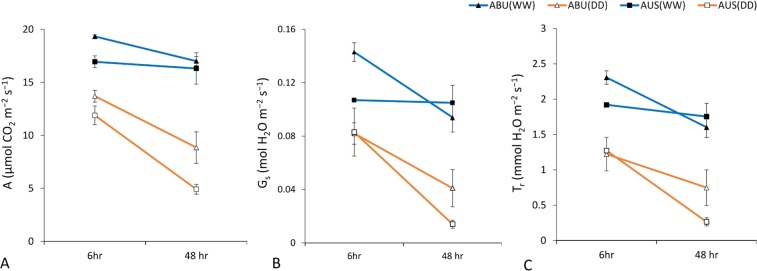
Effect of water stress on gas exchange parameters. (**A**) Net photosynthetic rate (**A**,**B**) Stomatal conductance (Gs) and (**C**) Transpiration rate (Tr) of the tolerant genotype, Abura (ABU) and susceptible genotype, AUS12671 (AUS) under control (WW) and stressed (DD) conditions.

Application of PEG stress resulted in a significant reduction (p < 0.05) in root length and root biomass in both ABU and AUS (Fig. 1). However, the tolerant genotype ABU suffered less, showed only about 14% reduction in root length and nearly 18% decline in root biomass under stress. On the other hand, % reduction in root length and biomass in the sensitive genotype AUS was about 4-fold and 2-fold higher, respectively, than the tolerant counterpart (Fig. 1). The contrasting genotypes also differ significantly in terms of gas exchange parameters under stress. Net photosynthetic rate (A), stomatal conductance (G_s_) and transpiration rate (T_r_) declined sharply due to stress in both the genotypes (Fig. 2). At 6 h of stress, ABU and AUS differ significantly (p < 0.05) only for net photosynthetic rate. However, with the more extended stress period (48 hours), marked differences were observed for all the three measured parameters, where the tolerant genotype had nearly 2-fold higher photosynthesis rate and 3-fold higher stomatal conductance and transpiration rate than the susceptible genotype (Fig. 2).

### Transcriptome quality and mapping statistics

A total of 216 Gb 150-bp paired-end (PE) reads were generated through deep whole transcriptome sequencing on a total of 24 root samples (2 genotypes × 2 treatments × 2 time-points × 3 replicates) from stressed and non-stressed (control) wheat seedling after quality control (Table S1). On average, about 60 million (M) clean reads were obtained from each library with sizes ranged from 7–15 Gb (Table S2). The reads were of high quality; nearly 98% and 95% of the clean reads had a quality score of Q20 and Q30, respectively. Additionally, the GC% of each library was about 57 (Tables S1 and S2).

More than three-quarters of the total reads were mapped to the wheat reference genome, including around 50% with a perfect match and about 21% with unique matches (Table S3). A multi-position match of 57.5 and 58.2% was observed in control (WW) and treated (DD) sample of ABU, respectively, whereas in the AUS genotype it was 54.8 and 56%, respectively. The total number of transcripts detected in each library ranged from 88,225 to 104,995, accounting for nearly 70% of all wheat genes (Table S2). Moreover, the genes with FPKM (transcript abundance of the gene) value of one or higher accounted for around 60% (Fig. S1) of all wheat genes.

### Differential gene expression in response to water deficit

**Figure 3:**
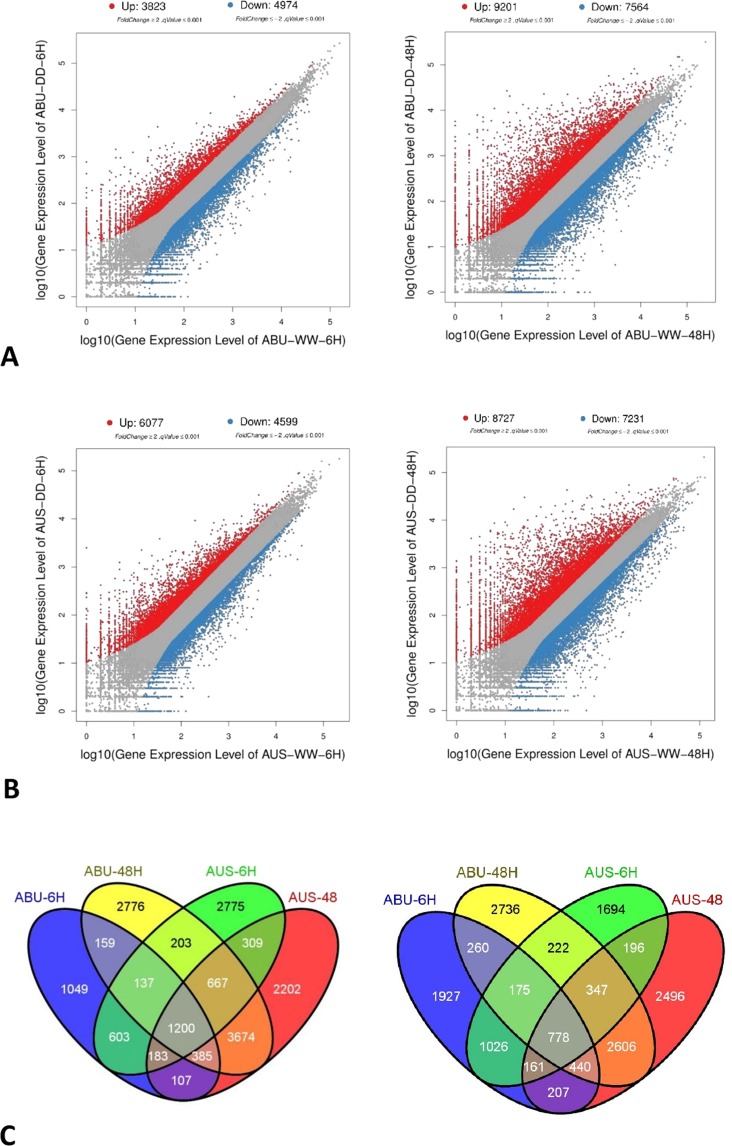
Differential gene expressions (control vs stressed) in the two genotypes, Abura (ABU) and AUS12671 (AUS) in two time-points of stress period (6 & 48 hours). (**A**) Scatter plots of gene expression in the tolerant genotype (ABU) at 6 h (left) and 48 h (right), showing that DEGs were greater in number at 48 h; (**B**) Scatter plots of gene expression in the susceptible genotype (AUS) at 6 h (left) and 48 h (right), showing that DEGs were greater in number at 48 h; and (**C**) Venn diagram showing the number of upregulated (left) and downregulated genes (right) in different combination of treatment-time points.

Transcriptome analysis of the two contrasting genotypes revealed significant differences in terms of initial and adaptive responses as evident from the gene expression pattern during early growth period water deficit. At 6 hours of stress imposition, a considerably higher number of genes (6077) were upregulated in the susceptible genotype AUS than the tolerant genotype ABU (Fig. 3A,B, Table S4). In contrast, the number of upregulated genes were slightly higher in tolerant genotype at 48 h stress period. However, a higher number of DEGs were observed at both 6 h and 48 h time point in the tolerant genotype. In general, the number of DEGs (both upregulated and downregulated) were higher when the stress period was longer (48 hr) (Fig. 3A,B, Table S4). To understand the adaptive mechanism in the tolerant and susceptible genotypes, particular attention was given to the genes whose differential regulations were genotype-specific and consistent in both 6 h and 48 h of stress. In the tolerant genotype, the number of genotype-specific genes that were consistently upregulated and downregulated across time points under stress were 159 and 260, respectively (Fig. 3C). In contrast, 309 and 196 genes were found to be consistently upregulated and downregulated, respectively, under stress in the susceptible genotype across time points (Fig. 3C).

### Gene ontology analysis of DEGs

**Figure 4:**
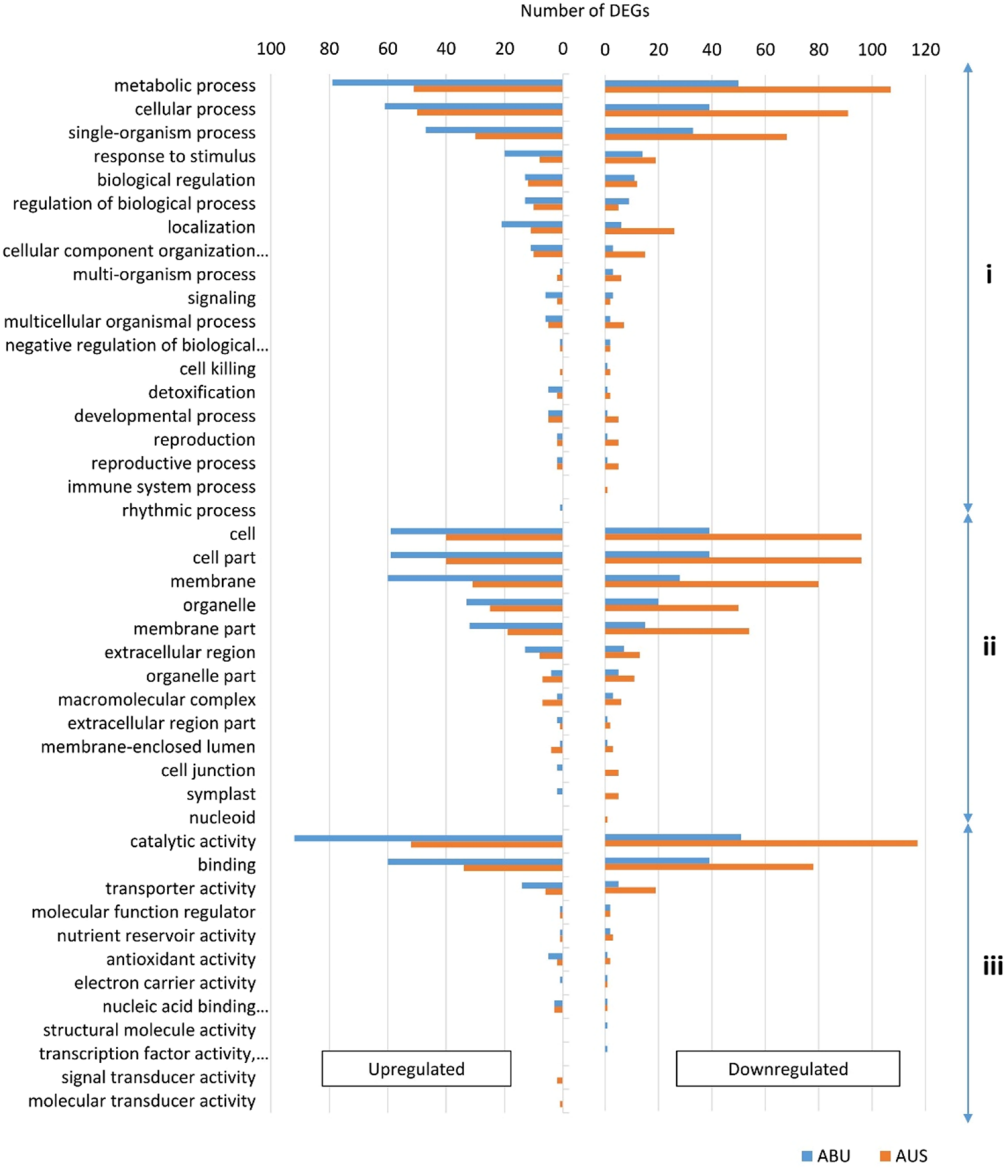
Number of differentially expressed genes (DEGs) enriched with different Gene Ontology (GO) terms in the tolerant genotype Abura (ABU, in blue) and susceptible genotype Aus12671 (AUS, in orange). The GO terms were grouped into three categories: (i) biological process, (ii) cellular component, and (iii) molecular function.

With genotype-specific and consistently expressed DEGs, upregulated and downregulated DEGs were functionally categorized into three principal categories: biological process, cellular component and molecular function (Fig. 4). Under biological process category, most of the genes were associated with the GO terms fell in the subcategories of “metabolic process”, “cellular process”, and “single organism process” in both the tolerant and susceptible genotypes. However, the most striking difference between the two genotypes was the number of upregulated and downregulated genes under each of these subcategories, with the susceptible genotype having nearly twice the number of upregulated genes (107, 91 and 68 respectively) than that (50, 39, and 33 respectively) in the tolerant genotype. In contrast, a considerably higher number of downregulated genes were associated with these three terms in the tolerant genotypes (79, 61, and 47 respectively) than in the susceptible genotype (51, 50 and 30 respectively). Moreover, the GO term “biological regulation” were enriched almost equally in both genotypes, but a considerably higher number of upregulated genes (26) were associated with the GO term “localization” in the susceptible genotype than in the tolerant genotype (Fig. 4). The term GO:0051179 or “localization” refers to any process in which a cell, a substance, or a cellular entity, such as a protein complex or organelle, is transported, tethered to or otherwise maintained in a specific location.

For cellular component categories, “cell”, “cell part”, “membrane”, “membrane part” and “organelle part” were the most frequent GO term subcategories in both the tolerant and susceptible genotypes with latter having considerably higher number of upregulated genes in each subcategory (96, 96, 80, 54 and 50, respectively) (Fig. 4). Noticeably, no downregulated genes were enriched with the term “membrane” in the susceptible genotype. Furthermore, upregulated genes associated with terms “cell junction” (5), and “symplast” (5) were assigned exclusively in the susceptible genotype, but in case of downregulated genes, they were enriched only in the tolerant genotype.

“Catalytic activity”, “binding” and “transporter activity” were the most abundant terms under the molecular function category. In the sensitive genotype, 117 and 78 upregulated genes were enriched with the term “catalytic activity” and “binding”, respectively, nearly 2-fold higher than the tolerant counterpart. However, in the case of downregulated genes, these two terms were more frequent in the tolerant genotype (92, and 60 respectively) than the susceptible genotype (52, 34 respectively). Moreover, only five upregulated genes gave the term “transporter activity” in the tolerant genotypes, about 4-times lesser than the sensitive genotype (19), whereas 14 and 6 downregulated genes were enriched with this term in the tolerant and susceptible genotypes, respectively (Fig. 4).

### KEGG pathway enrichment analysis

**Figure 5:**
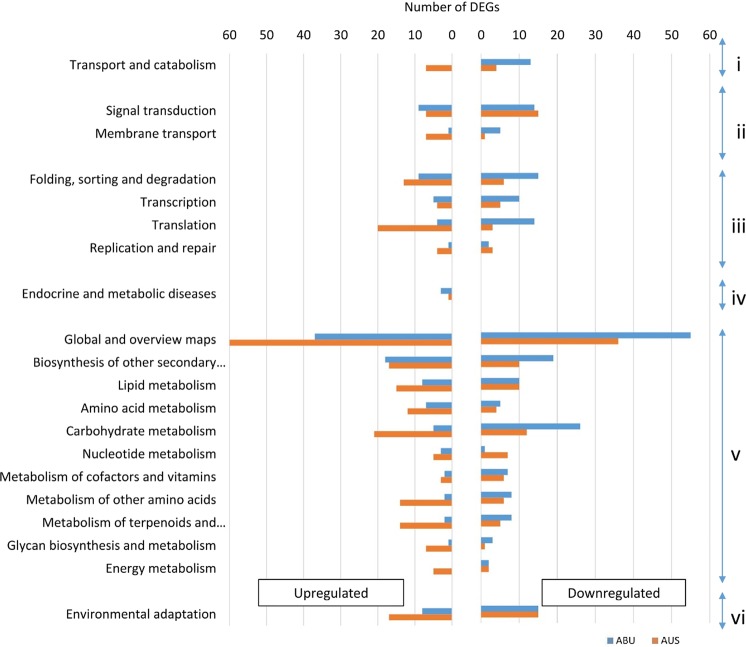
Number of differentially expressed genes (DEGs) enriched with different KEGG pathways in the tolerant genotype Abura (ABU, in blue) and susceptible genotype Aus12671 (AUS, in orange). Pathways were grouped into six categories (i) cellular process, (ii) environmental information processing, (iii) genetic information processing, (iv) disease-related processes, (v) metabolism, and (vi) organismal systems.

Pathway enrichment analysis assigned genotype-specific and consistently expressed DEGs across time points to different pathways belonging to six major categories, including “environmental information processing”, “genetic information processing”, “cellular processes”, “metabolism”, “organismal systems’, and “disease-related” (Fig. 5). Interestingly, no enrichment of “transport and catabolism” pathway of the cellular process was observed in the tolerant genotype. Although the “signal transduction” and “transcription” pathways of AUS and ABU were upregulated to the similar frequency, AUS showed 4–5 fold enrichment of upregulated genes influencing “translation” and “replication and repair” related pathways. In both the tolerant and susceptible genotypes about 60–70% genes accounted for pathways related to metabolism categories. However, except for pathways related to “biosynthesis of secondary metabolites” and “metabolism of cofactors and vitamins”, all the pathways of this category were upregulated largely in the sensitive genotype than the tolerant genotype. Similarly, the number of upregulated genes annotated in “environmental adaptation” was 2-fold higher in the sensitive than the tolerant genotype. However, this pathway was downregulated to the same degree in both genotypes. (Fig. 5).

Among the downregulated “metabolism” pathways, the top three pathways, “global and overview maps”, “carbohydrate metabolism” and “biosynthesis of secondary metabolites”, were enriched to a greater extent in the tolerant genotype (Fig. 5). In contrast, no other pathway of this category accounted for significant changes in the number of downregulated genes between AUS and ABU, except for “nucleotide metabolism” which showed a seven-fold higher enrichment in the susceptible genotype. Whereas, “translation”, “transcription”, “folding, sorting and degradation”, “membrane transport” and “transport and catabolism” were downregulated with a higher frequency in the tolerant genotype than the susceptible genotype.

### Transcription factor (TF) encoding genes

**Figure 6:**
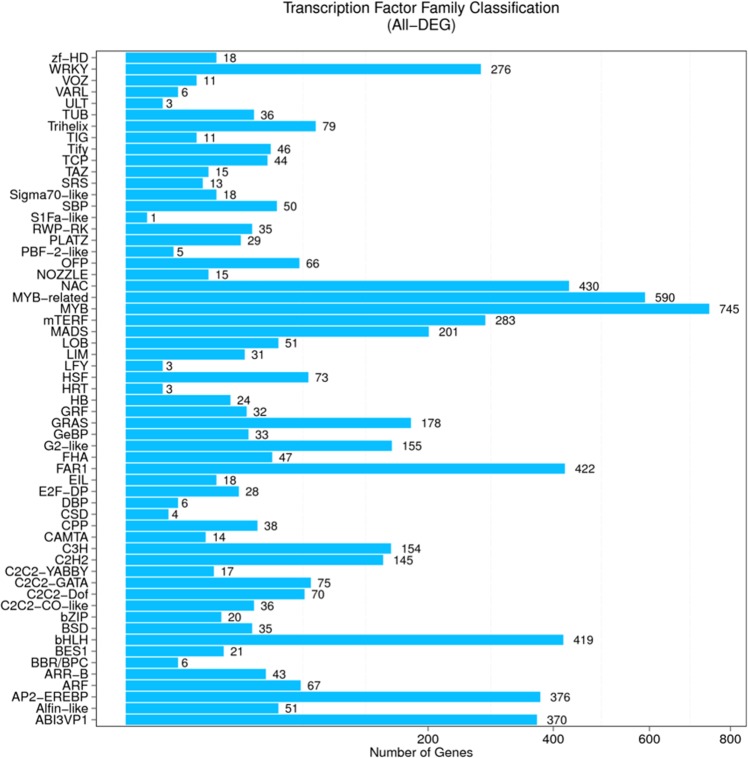
Distribution of DEGs in different Transcript factors (TF) families considering the whole transcriptome.

**Figure 7:**
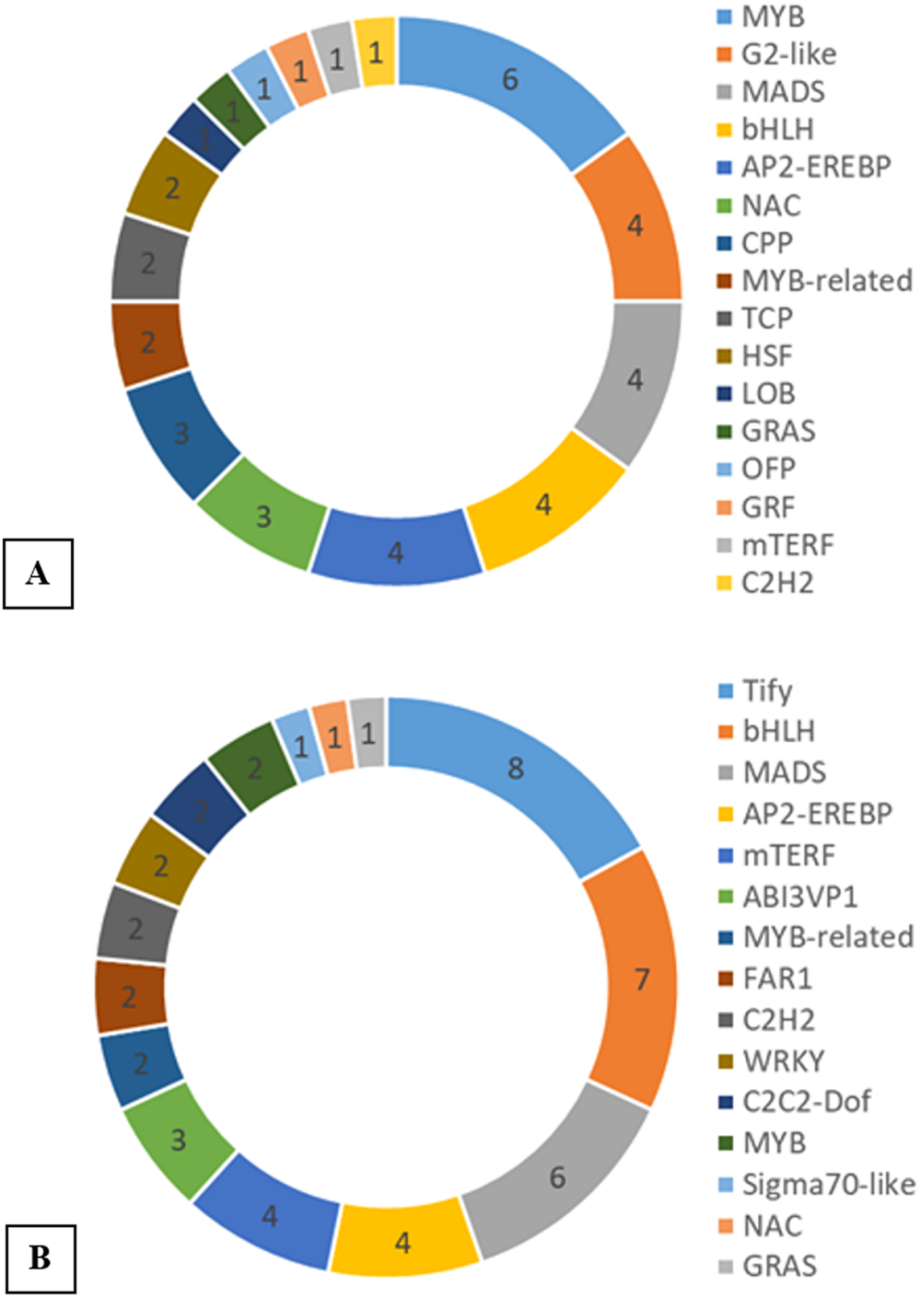
Transcription factors encoding DEGs (genotype-specific and consistently expressed) and their distributions. (**A**) Distribution of the consistently expressed and tolerant genotype-specific DEGs encoding different TFs; (**B**) Distribution of the consistently expressed and susceptible genotype-specific DEGs encoding different TFs.

Transcription factors play a vital role as molecular switches controlling the expression of specific genes and regulating plant growth and development under certain environmental conditions. Extensive database searches of all the DEGs predicted a total 6,088 TFs to be differentially expressed. These TFs could be grouped into 60 families. MYB and MYB-related TFs were the highest among the identified TF families. Other TFs with a larger number of encoding genes (~400) were NAC, FAR1, and BHLH (Fig. 6). Sixteen TF families encoded by 40 DEGs were expressed uniquely in the tolerant genotype across time points. These were MYB, G2-like, MADS, bHLH, AP2-EREBP, NAC, CPP, MYB-related, TCP, HSF, LOB, GRAS, OFP, GRF, mTERF, and C2H2 (Fig. 7A). Of them, MYB were encoded by the highest number of DEGs (6). In contrast, Tify, Bhlh, MADS, AP2-EREBP and Mterf were the mostly encoded TFs in the sensitive genotype (Fig. 7B).

## Discussion

Several previous studies indicated that plants subjected to water deficit exhibit wide ranges of responses including molecular expression^27^, biochemical processes^28^ and involving various genes and pathways^29^. In this study, transcriptomic changes in wheat roots during early seedling growth stages (Zadoks scale 11–12) were investigated in contrasting genotypes differing in root morphology under water deficit condition. It was hypothesised that these differences, when linked with differences in gene expressions between the contrasting genotypes, would allow us to explore the underlying mechanism, identify the important genes, transcription factors and complex pathways that play critical roles for water deficit tolerance at the early growth stage in wheat.

We found that relatively longer roots of the tolerant genotype ABU assisted in comparatively higher transpiration and stomatal conductance, subsequently better photosynthesis leading to higher root biomass than the susceptible genotype AUS under stress. Transcriptomic analysis of the two contrasting genotypes also revealed that despite subjecting to the same extent of water deficit, the responses differed significantly between the tolerant and the susceptible genotypes in terms of the number of genes expressed (Fig. 3), pathways (Fig. 5) and transcription factors (Fig. 7) involved. A significantly higher number of genotype-specific and consistently expressed DEGs between stressed and non-stressed conditions were detected in the susceptible genotype AUS than those in the tolerant genotype ABU. Fracasso, *et al*.^30^ also reported higher expression of drought-related genes in the susceptible genotype than the tolerant genotype. The predominance of upregulation of genes under stress, especially for the metabolism related ones, such as carbohydrate, lipid, amino acid and terpenoids (Fig. 4), could be an indication of energy-consuming approach of the susceptible genotype in adaptation to water deficit. In contrast, downregulation of metabolism related genes was more prominent in the tolerant genotype along with downregulating the synthesis of secondary metabolites suggesting that the tolerant genotype adopted an energy-saving approach in response to the stress imposition.

Further investigation of the DEGS that were uniquely upregulated in the tolerant genotype and their related KEGG pathways revealed that except two leading pathways of the tolerant genotype: (1) metabolic pathways (pathway ID ko01100) and (2) pathways related to biosynthesis of secondary metabolites (ko01110), other pathways including those related to flavonoid biosynthesis (ko00941), plant hormone signal transduction (ko04075), and phenylpropanoid biosynthesis (ko00940) contained relatively higher number of DEGs (Table S5). Previous studies reported that drought led to accumulation of reactive oxygen species (ROS) which causes oxidative stress in plants^31^. Secondary metabolites, such as phenylpropanoids and flavonoids, act as antioxidants defense to protect plants from damaging effects of oxidative stress^32,33^. In wheat and barley, accumulation of phenylpropanoids and flavonoid was observed in response to drought stress together with upregulation of genes involved in the phenylpropanoids and flavonoid biosynthetic pathway^34,35^. In this study, we found a total 13 genes, seven for the flavonoid biosynthesis pathway and six for the phenylpropanoid biosynthesis pathway, that were exclusively upregulated in the tolerant genotype under stress (Table S5). In addition, another set of seven upregulated genes that were unique to tolerant genotype across time points were found to be involved in plant signal transduction pathways, two of which encoding SAUR (small auxin up-regulated RNA) protein responsible for cell enlargement and plant growth (Table S5). Stortenbeker and Bemer^36^ reviewed the role of SAUR gene family for adaptation of plant growth and development. Li, *et al*.^37^ reported that SAUR proteins promoted plant growth in Arabidopsis.

It was reported that MYB transcription factors control many crucial biological processes under limited water condition^38^. Our study also revealed that MYB TF families were highly enriched in tolerant genotype ABU across time points. MYB96, a modulator of the ABA signalling pathway in Arabidopsis, was reported to be involved in restricting initial lateral root elongation so that primary root growth was maintained and accessed deeper soil moisture under stress^17^. RNA-seq analysis by Zhao, *et al*.^39^ indicated TaMYB31, an ortholog of AtMYB96, functioned as a positive regulator of drought resistance through up-regulation of wax biosynthesis genes and drought-responsive genes. Another MYB transcription factors, TaMYB sdu1 was also reported to be upregulated in roots of PEG-6000 treated wheat seedlings^40^. Besides MYBs, several NAC transcription factors were also consistently expressed in tolerant genotype ABU. In PEG-treated wheat seedlings, overexpression of TaRNAC1, a predominantly root-expressed NAC transcription factor, conferred increased root length, biomass and dehydration tolerance^41^. In another study, it was reported that under water-limited conditions up-regulation of TaNAC69-1 acted as a transcriptional repressor of the root growth-inhibitory genes, thus enabling root elongation, enhancing biomass in wheat under stressed condition^42^. All the above-mentioned reports suggest that MYB and NAC TFs might have played a crucial role in root growth, root system proliferation and overall biomass in the tolerant genotypes under stress.

In Summary, high-throughput RNA-seq technology was employed in this study to characterize the root transcriptome of the contrasting genotypes under early seedling stage water deficit. The differential response of the two genotypes was evident from their root morphological features and physiological measurements under stress. The sensitive genotype AUS suffered significantly due to early-stage water deficit. These were further characterized by the root transcriptome analysis where sensitive genotype showed a higher number of DEGs. Upregulation of DEGs related to all major key metabolic pathways suggested the hypersensitive response of the susceptible genotype AUS under stress, whereas the tolerant genotype was generally less affected. Additionally, not only the secondary metabolites such as phenylpropanoids and flavonoid, but also the transcription factors MYB, NAC might also be associated with the stress adaptation strategies in the tolerant genotype.

## Materials and methods

### Plant materials, growth condition, treatment application and tissue sampling

Two genotypes of bread wheat, Abura and AUS 12671 having contrasting feature of % root length reduction under water deficit were selected for this study. Healthy, uniform-sized, surface-sterilized seeds of those two genotypes were germinated on soaked filter paper and grown in a hydroponic system (pH set at 5.7–5.9) housed in a controlled plant growth chamber (temperature of 22 ± 2 °C with 16:8 hours light:dark cycle, light intensity of 300 µmol m^−2^s^−1^) at the University of Western Australia. Twenty seedlings from each genotype were grown in half-strength Hoagland solution for the initial ten days after germination, and afterwards, stress treatments were applied by supplementing the growing medium with 20% PEG6000 (osmotic potential of −0.50 ± 2 MPa), whereas controlled plants received no supplement. For transcriptomic study, roots were collected from both the genotypes of control and stress treatment at 6 hours and 48 hours of stress imposition in three biological replicates, with each biological replicate containing roots of three randomly selected seedlings. Collected root samples were frozen immediately in liquid nitrogen and stored at −80 °C until RNA extraction. Before root sampling, gas exchange parameters (net photosynthesis rate, transpiration rate, stomatal conductance) were also measured using a portable photosynthesis system (LI-6400, Li-COR Inc., Lincoln, NE, USA) at 6 hours and 48 hours of stress imposition. The system was adjusted with the following settings: 2 × 3 cm EB (energy balance) opaque cuvette, block temperature (20 °C), CO_2_ concentration (400 µmols^−1^) and LED light intensity (1000 µmolm^−2^ s^−1^). Seedlings were then grown up to seven days of stress period and harvested on the seventh day to examine the morphological differences in root traits. Root length, per sent root length reduction under stress, and dry root biomass (oven-dried at 65 °C for 72 hours) were measured for each genotype-treatment combination.

### Total RNA extraction, library preparation and sequencing

Total RNA was extracted from 24 samples (2 genotypes × 2 treatments × 2 time-points × 3 replicates) using RNeasy Plus Plant mini kit (Qiagen) with an on-column DNase treatment. The concentration of the extracted RNA was checked by NanoDrop 2000 (ND-2000, Thermo Fisher Scientific, Inc., CA, USA), and quality was checked by 1% (w/v) denatured gel electrophoresis and Agilent 2100 Bioanalyzer (Agilent Technologies, Inc., CA, USA). The samples were then sent to the Beijing Genomics Institute (BGI), China, for sequencing. In short, mRNAs were isolated from total RNA with oligo (dT) method and fragmented, which were then used for cDNA synthesis. 150-bp paired-end sequencing libraries were prepared and sequenced using HiSeq X Ten (Illumina, San Diego, USA) according to manufacturer’s standard protocols. Raw sequencing data were processed by removing adapters, reads with unknown bases (N’s > 5%) and low quality (% bases with Phred score <15 is higher than 20%) to generate “clean data” as FastQ files using SOAPnuke software (version: v1.5.2)^43^. Q20, Q30 and GC contents of the clean data were also calculated. All downstream analyses were performed on clean data of those 24 libraries, which are publicly available at the National Centre for Biotechnology Information (NCBI) website with the accession or bioproject number of PRJNA521521.

### Differential gene expression analysis

Processed high-quality paired-end reads from each library were mapped to the bread wheat reference (ftp://ftp.ensemblgenomes.org/pub/plants/release-39/fasta/triticum_aestivum/dna/) sequence using HISAT2 (Hierarchical Indexing for Spliced Alignment of Transcripts) software, version v2.0.4^44^. Reads were then aligned to the reference sequence using Bowtie2^45^, and the gene expression level was calculated using RSEM (RNA-Seq by Expectation Maximization) software Version: v1.2.12^46^ with default parameter. Pearson correlation between all samples was calculated using ‘cor’ function in R software. DEGs were detected with DEGseq as described in Wang, *et al*.^47^ with the following parameters: Fold Change (control vs stressed) >= 2 and Adjusted P-value < = 0.001.

### Functional annotations, GO enrichment and pathway analysis

Gene ontology (GO) classification and functional enrichment analysis were performed using the hypergeometric test (phyper), with the selected DEGs. DEGs with false discovery rate (FDR) not larger than 0.01 were defined as significantly enriched. With the KEGG annotation result, DEGs were classified according to official classification, and pathway functional enrichment was also performed using phyper. FDR for each p-value was calculated.

Getorf^48^ was used to find open reading frame (ORF) of each DEG. ORFs were then aligned to transcription factor (TF) domains from PlntfDB using hmmsearch^49^ to identify TF encoding genes from the selected DEGs.

## Supplementary information

Supplementary information
