# Supplementary material for: Root transcriptome profiling of contrasting wheat genotypes provides an insight to their adaptive strategies to water deficit
Source: Sci Rep. 2020 Mar 17;10:4854. doi: 10.1038/s41598-020-61680-1 (PMC7078264; doi:10.1038/s41598-020-61680-1)
Supplement: Supplementary file 1 — Supplementary information [file 41598_2020_61680_MOESM1_ESM.pdf]

Root transcriptome profiling of contrasting wheat genotypes provides an insight to their adaptive strategies to water deficit

Md Sultan Mia[1,2,3], Hui Liu[1,2*], Xingyi Wang[1,2], Chi Zhang[4] and Guijun Yan[1,2*]

[1]UWA School of Agriculture and Environment, Faculty of Science, The University of Western Australia, Perth, WA, Australia

[2]The UWA Institute of Agriculture, The University of Western Australia, Perth, WA, Australia

[3]Department of Plant Breeding, Bangladesh Agricultural Research Institute, Gazipur, Bangladesh

4 Beijing Genomics Institute-Shenzhen, Shenzhen 518083, China

***Correspondence:**

Hui Liu hui.liu@uwa.edu.au

Guijun Yan guijun.yan@uwa.edu.au

Table S1: Summary of the transcriptome sequencing and quality

	Sum of Total Clean Reads(M)	Sum of Total Clean Bases(Gb)	Mean Q20 (%)	Mean Q30 (%)	Mean GC (%)
**ABU**	**768.58**	**115.3**	**98.32**	**94.83**	**57**
WS	371.08	55.66	98.32	94.84	57
WW	397.5	59.64	98.32	94.83	57
**AUS**	**673.65**	**101.05**	**98.38**	**95.01**	**57**
DDWS	318.93	47.84	98.30	94.75	57
WW	354.72	53.21	98.46	95.27	56
**Grand Total**	**1442.23**	**216.35**	**98.35**	**94.92**	**57**

ABU: The tolerant genotype, Abura
AUS: The susceptible genotype, AUS12671
DD: PEG-treated water-stressed condition
WW: Well-watered or control condition

Table S2: Sample-wise read quality and mapping statistics of the whole transcriptome of Abura and AUS12671

Sample	Reads (million)	Bases (Gb)	Q20 (%)	Q30 (%)	Number of genes mapped	Number of transcripts mapped
1-AUS-WW-6H	64	10	99	96	73424	95594
2-AUS-WW-6H	67	10	99	96	76042	98792
3-AUS-WW-6H	53	8	98	95	76094	97628
4-AUS-WW-48H	60	9	98	95	75731	96845
5-AUS-WW-48H	65	10	98	95	74751	95278
6-AUS-WW-48H	45	7	98	95	75641	98734
7-AUS-DD-6H	56	8	98	95	73347	91699
8-AUS-DD-6H	58	9	98	95	77330	102107
9-AUS-DD-6H	51	8	98	95	76820	99141
10-AUS-DD-48H	54	8	98	94	76378	97658
11-AUS-DD-48H	53	8	98	95	75012	94935
12-AUS-DD-48H	47	7	98	95	77751	101532
13-ABU-WW-6H	57	9	98	95	77437	101704
14-ABU-WW-6H	48	7	98	95	76672	100306
15-ABU-WW-6H	64	10	98	95	78311	103907
16-ABU-WW-48H	102	15	98	95	77709	101043
17-ABU-WW-48H	73	11	98	95	75331	96224
18-ABU-WW-48H	54	8	98	95	77428	102013
19-ABU-DD-6H	54	8	98	95	71758	88225
20-ABU-DD-6H	78	12	98	95	76959	100075
21-ABU-DD-6H	55	8	98	95	75763	97963
22-ABU-DD-48H	69	10	98	95	79372	104995
23-ABU-DD-48H	56	8	98	95	74187	92919
24-ABU-DD-48H	59	9	98	95	74062	92480
**Average**	**60**	**9**	**98**	**95**	**75971**	**97992**

Table S3: Mapping summary of the transcriptome

Reads	ABU		AUS	
	DD (%)	WW (%)	DD (%)	WW (%)
Total Mapped	79.2	78.1	77.0	75.6
Perfect Match	52.2	51.5	49.5	48.0
Mismatch	27.0	26.5	27.5	27.6
Unique Match	20.9	20.6	21.0	20.7
Multi-position Match	58.2	57.5	56.0	54.8
Unmapped	20.8	21.9	23.0	24.4

Table S4: Differentially expressed gene counts in contrasting genotypes at different time points of stress

Genotype		6h	48h
ABU (WWvsDD)	Up	3823	9201
	Down	4974	7564
AUS (WWvsDD)	Up	6077	8727
	Down	4599	7231

Table S5: Upregulated genes related to KEGG pathway in the tolerant genotype

Pathway	DEGs with pathway annotation (100)	P-value	Q value	Pathway ID	Level 1	Level 2
**Flavonoid biosynthesis**	7 (7%)	2.49E-05	0.001347	ko00941	Metabolism	Biosynthesis of other secondary metabolites
Biosynthesis of secondary metabolites	27 (27%)	0.000277	0.00749	ko01110	Metabolism	Global and overview maps
AGE-RAGE signalling pathway in diabetic complications	3 (3%)	0.002981	0.051689	ko04933	Human Diseases	Endocrine and metabolic diseases
Isoquinoline alkaloid biosynthesis	3 (3%)	0.003829	0.051689	ko00950	Metabolism	Biosynthesis of other secondary metabolites
Isoflavonoid biosynthesis	3 (3%)	0.009054	0.087111	ko00943	Metabolism	Biosynthesis of other secondary metabolites
Tyrosine metabolism	3 (3%)	0.009679	0.087111	ko00350	Metabolism	Amino acid metabolism
Metabolic pathways	31 (31%)	0.023747	0.183192	ko01100	Metabolism	Global and overview maps
Stilbenoid, diarylheptanoid and gingerol biosynthesis	3 (3%)	0.028216	0.190455	ko00945	Metabolism	Biosynthesis of other secondary metabolites
Benzoxazinoid biosynthesis	2 (2%)	0.050663	0.303978	ko00402	Metabolism	Biosynthesis of other secondary metabolites
Cutin, suberine and wax biosynthesis	2 (2%)	0.091412	0.425833	ko00073	Metabolism	Lipid metabolism
**Plant hormone signal transduction**	7 (7%)	0.093392	0.425833	ko04075	Environmental Information Processing	Signal transduction
Phenylalanine metabolism	2 (2%)	0.095481	0.425833	ko00360	Metabolism	Amino acid metabolism
Steroid biosynthesis	2 (2%)	0.102515	0.425833	ko00100	Metabolism	Lipid metabolism
**Phenylpropanoid biosynthesis**	6 (6%)	0.111964	0.431862	ko00940	Metabolism	Biosynthesis of other secondary metabolites
Anthocyanin biosynthesis	1 (1%)	0.171989	0.578239	ko00942	Metabolism	Biosynthesis of other secondary metabolites
Flavone and flavonol biosynthesis	1 (1%)	0.182932	0.578239	ko00944	Metabolism	Biosynthesis of other secondary metabolites
Tryptophan metabolism	2 (2%)	0.193795	0.578239	ko00380	Metabolism	Amino acid metabolism
Circadian rhythm - plant	2 (2%)	0.196675	0.578239	ko04712	Organismal Systems	Environmental adaptation
Tropane, piperidine and pyridine alkaloid biosynthesis	1 (1%)	0.244642	0.578239	ko00960	Metabolism	Biosynthesis of other secondary metabolites
RNA degradation	3 (3%)	0.260082	0.578239	ko03018	Genetic Information Processing	Folding, sorting and degradation
Phenylalanine, tyrosine and tryptophan biosynthesis	1 (1%)	0.273259	0.578239	ko00400	Metabolism	Amino acid metabolism
Ubiquitin mediated proteolysis	3 (3%)	0.282681	0.578239	ko04120	Genetic Information Processing	Folding, sorting and degradation
Pentose and glucuronate interconversions	2 (2%)	0.297781	0.578239	ko00040	Metabolism	Carbohydrate metabolism
beta-Alanine metabolism	1 (1%)	0.316466	0.578239	ko00410	Metabolism	Metabolism of other amino acids
Ether lipid metabolism	1 (1%)	0.325517	0.578239	ko00565	Metabolism	Lipid metabolism
Linoleic acid metabolism	1 (1%)	0.329104	0.578239	ko00591	Metabolism	Lipid metabolism

Sesquiterpenoid and triterpenoid biosynthesis	1 (1%)	0.339752	0.578239	ko00909	Metabolism	Metabolism of terpenoids and polyketides
Pyrimidine metabolism	3 (3%)	0.340657	0.578239	ko00240	Metabolism	Nucleotide metabolism
Basal transcription factors	1 (1%)	0.341511	0.578239	ko03022	Genetic Information Processing	Transcription
Protein processing in endoplasmic reticulum	5 (5%)	0.356991	0.578239	ko04141	Genetic Information Processing	Folding, sorting and degradation
Purine metabolism	3 (3%)	0.367826	0.578239	ko00230	Metabolism	Nucleotide metabolism
MAPK signaling pathway - plant	4 (4%)	0.369109	0.578239	ko04016	Environmental Information Processing	Signal transduction
Spliceosome	3 (3%)	0.374684	0.578239	ko03040	Genetic Information Processing	Transcription
RNA polymerase	2 (2%)	0.377871	0.578239	ko03020	Genetic Information Processing	Transcription
Ubiquinone and other terpenoid-quinone biosynthesis	1 (1%)	0.383171	0.578239	ko00130	Metabolism	Metabolism of cofactors and vitamins
Arachidonic acid metabolism	1 (1%)	0.394587	0.578239	ko00590	Metabolism	Lipid metabolism
Diterpenoid biosynthesis	1 (1%)	0.396201	0.578239	ko00904	Metabolism	Metabolism of terpenoids and polyketides
RNA transport	3 (3%)	0.422223	0.600001	ko03013	Genetic Information Processing	Translation
N-Glycan biosynthesis	1 (1%)	0.45081	0.608511	ko00510	Metabolism	Glycan biosynthesis and metabolism
Glycine, serine and threonine metabolism	1 (1%)	0.455194	0.608511	ko00260	Metabolism	Amino acid metabolism
Amino sugar and nucleotide sugar metabolism	2 (2%)	0.462018	0.608511	ko00520	Metabolism	Carbohydrate metabolism
Plant-pathogen interaction	6 (6%)	0.497803	0.618859	ko04626	Organismal Systems	Environmental adaptation
Terpenoid backbone biosynthesis	1 (1%)	0.504516	0.618859	ko00900	Metabolism	Metabolism of terpenoids and polyketides
mRNA surveillance pathway	3 (3%)	0.513527	0.618859	ko03015	Genetic Information Processing	Translation
Porphyrin and chlorophyll metabolism	1 (1%)	0.519525	0.618859	ko00860	Metabolism	Metabolism of cofactors and vitamins
Indole alkaloid biosynthesis	1 (1%)	0.527176	0.618859	ko00901	Metabolism	Biosynthesis of other secondary metabolites
Glycerophospholipid metabolism	1 (1%)	0.576894	0.654078	ko00564	Metabolism	Lipid metabolism
Homologous recombination	1 (1%)	0.581403	0.654078	ko03440	Genetic Information Processing	Replication and repair
ABC transporters	1 (1%)	0.605896	0.667722	ko02010	Environmental Information Processing	Membrane transport
Ascorbate and aldarate metabolism	1 (1%)	0.662229	0.715207	ko00053	Metabolism	Carbohydrate metabolism
Glycerolipid metabolism	1 (1%)	0.752733	0.797012	ko00561	Metabolism	Lipid metabolism
Cyanoamino acid metabolism	1 (1%)	0.797996	0.828688	ko00460	Metabolism	Metabolism of other amino acids
Biosynthesis of amino acids	1 (1%)	0.936693	0.936952	ko01230	Metabolism	Global and overview maps
Starch and sucrose metabolism	1 (1%)	0.936952	0.936952	ko00500	Metabolism	Carbohydrate metabolism

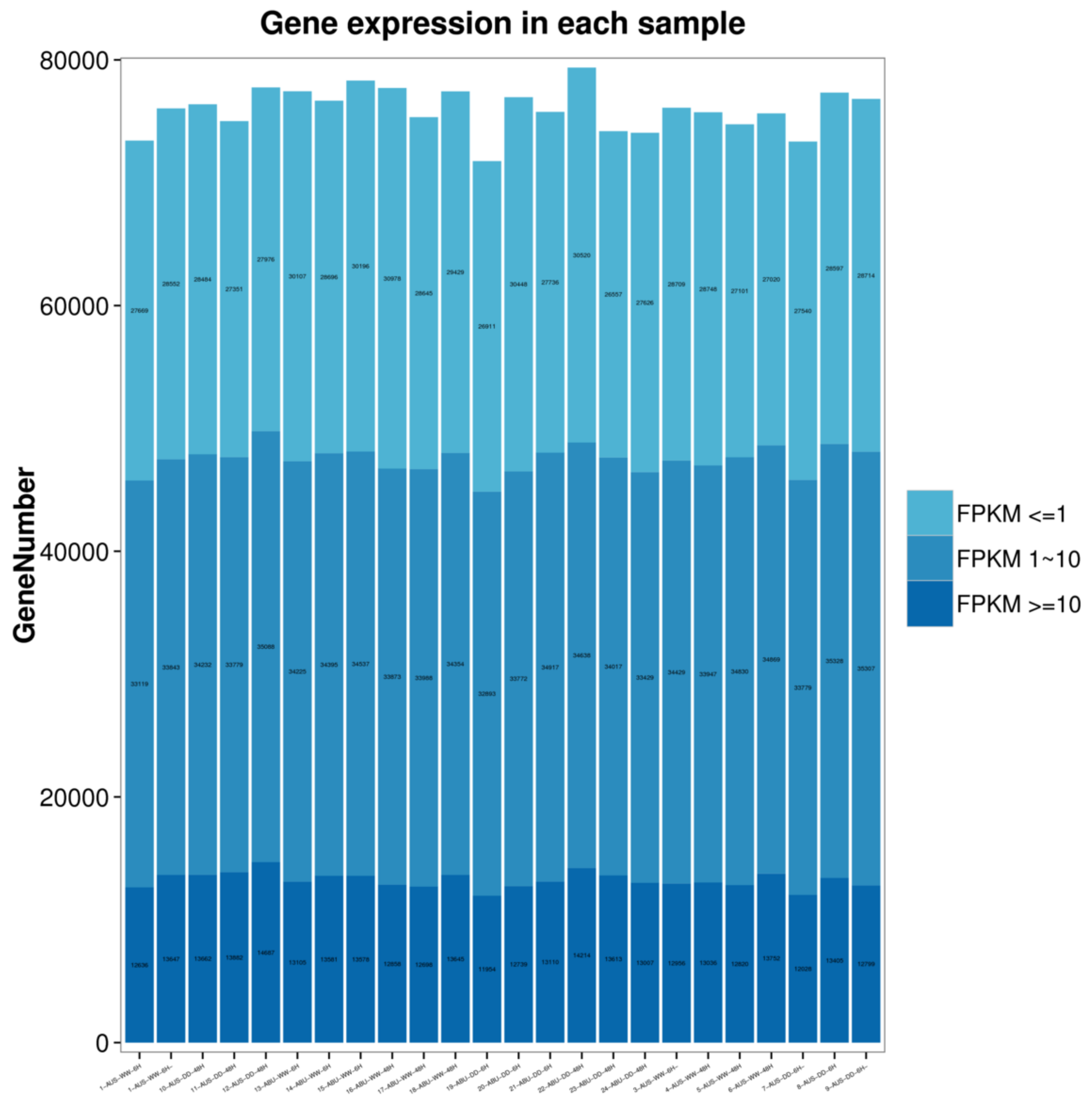

Figure S1: Number of genes with different FPKM (Fragments Per Kilobase of transcript Per Million) value in the 24 RNA-seq samples
